# Teaching and learning medical humanities in medical school: a student's perspective on professional practice curriculum in Australia

**DOI:** 10.1007/s40592-025-00258-x

**Published:** 2025-06-30

**Authors:** Matthew Melhem, Vinita Rane, Charlotte Denniston, George P. Drewett

**Affiliations:** 1 Melbourne Medical School, University of Melbourne, Parkville, Victoria Australia; 2 The Northern Hospital, Epping, Victoria Australia

**Keywords:** Professional practice, Medical humanities, Medical education, Curriculum development, Student perspective

## Abstract

The growing integration of the medical humanities in medical school curricula highlights its importance in the development of culturally safe, patient-centred clinicians. Internationally, medical schools attempt to increase student engagement through course electives, different modes of assessment and diverse content delivery. At the University of Melbourne, the Professional Practice program aims to provide an easy, engaging way of exposing students to the medical humanities, including reflective practice, collaborative practice, leadership, advocacy, professional identity formation, medical ethics and law. However, students’ perceptions of the medical humanities may prevent desired outcomes from being reached. We discuss the student experience of the Professional Practice curriculum through a collaboration between a student, tutor and course designers focusing on student engagement and perspectives of the program. Overall, students felt uncomfortable with the flexibility and ambiguity of the medical humanities when compared to the rigidity of biomedical knowledge. Additionally, modes of assessment typically used in the humanities such as reflective writing were found to be unpopular. Students’ involvement in the co-facilitation of classes helped develop communication skills and leadership but overall participation was still dependent on individual factors. Ultimately, the medical school and student body must work together to develop a medical humanities curriculum that is both complementary and viewed as of equal importance to the clinical curriculum.

## Introduction

Medical humanities education aims to mould medical students into doctors that see patients as more than a collection of symptoms (Shapiro et al. 2009; Batistatou et al. 2010). By drawing from multiple disciplines including anthropology, the arts, philosophy and literature, the medical humanities instil themes of ambiguity, nuance and uncertainty that are more reflective of the human condition (Bleakley 2023). This not only improves patient outcomes but encourages students to critically reflect on their interactions with the healthcare system, aiding professional and personal development. Traditionally, medical schools have focused on clinical and biomedical knowledge as the pillar of medical education (Assing Hvidt et al. 2022). However, there has been a shift towards integrating the medical humanities into medical school curricula (Wachtler et al. 2006). The Australian Medical Council (AMC) highlights the importance of practicing “whole-person care” which not only considers physical needs, but also “emotional, social, economic, environmental, cultural and spiritual needs” (AMC 2023).

The provision of the medical humanities in medical school curricula is highly variable (Howick et al. 2022). Since there is no prescribed structure, this allows for different interpretations of how best to instil humanistic values in students. In particular, the University of Melbourne Medical School aims to embed these values in students through its Professional Practice (PP) program. However, medical students’ perception of the humanities can be at odds with its aims and outcomes (Assing Hvidt et al. 2022). Students often enter medical school with the idea that the humanities are inferior and inconsequential when compared to clinical knowledge (Assing Hvidt et al. 2022). With time management pressures and biomedical study forming the bulk of the degree, addressing the humanities can be seen by students as superfluous and irrelevant.

This notable discordance between student engagement and the utility of the medical humanities has prompted questions over the effectiveness of these initiatives (Smydra et al. 2022). We explored the student experience of this through the primary author’s engagement with the PP curriculum at the University of Melbourne.

The student reflection will centre around two core ideas: Firstly, we explore the diversity of student perspectives of the integrated PP program; from futility to appreciation, opinions of the course are shared through a discussion of the curriculum’s reputation, subject matter and assessments. Secondly, the concept of student-facilitated content delivery is explored with respect to co-facilitation of tutorials. This original initiative encouraged students to be actively engaged in teaching, discussion and leadership. Overall, this article aims to provide new insights into current student attitudes towards learning the medical humanities in medical school, as well as the value of student co-facilitation to increase student participation and immersion.

## Medical humanities in medical school

The medical humanities form an important part of medical school education, encouraging students to incorporate a psychological and social lens to biomedical thinking. This allows for a deeper understanding of the dimensions underpinning healthcare whilst also harnessing the skills and attitudes to be a well-rounded clinician (Carr et al. 2021). As a result, this helps build effective doctor-patient relationships which can improve understanding of symptoms and adherence to treatment plans (Zhang 2024). Other benefits include better grades, greater empathy and reduced burnout (Kopelman 1995; Rolfe et al. 1995; Gordon 2005). In particular, internship is a difficult transition period where rates of emotional burnout significantly increase (Willcock et al. 2004). Thus, teaching the medical humanities acts as a protective mechanism through an emphasis on reflective practice, which has been shown to increase self-awareness and insight into internal biases (Gordon 2005). However, there is great difficulty in developing these skills due to the subjective nature of the humanities (Thacker et al. 2022). Since students are attuned to the objectivity of biomedicine, introducing a nuanced approach to clinical medicine may be disorienting. Consequently, student engagement is dependent on presenting material in ways that do not alienate but provide space to sit with uncertainty. Otherwise, students may find it uncomfortable and unhelpful.

Incorporating medical humanities into the medical school curriculum is common (Howick et al. 2022). Many medical schools provide both compulsory classes and elective subjects that integrate the arts, philosophy and sociology into the main coursework (Howick et al. 2022). The transition from a paternalistic to a patient-centred model of healthcare has prompted the inclusion of medical humanities into the medical degree. However, since the Flexner report in 1910, medical school curricula have been slow to adapt to the evolving pedagogy on an effective medical humanities curriculum (Duffy 2011; Bleakley 2015; de Oliveira et al. 2024). Flexner’s report was a transformative agenda that sought to overhaul medical school curricula by emphasising biomedical science, shifting education away from a humanistic approach (Bleakley 2015). The lasting effects of the report may have conditioned students and medical courses alike to prioritise scientific knowledge and devalue the humanities (Duffy 2011). Despite this, current thought has evolved from Flexner and towards a curriculum that harmoniously blends the medical humanities and clinical medicine (Bleakley 2015). Nevertheless, it is not enough to include the medical humanities into medical school; it is also important to mould its teachings to the modern age in order to produce effective practitioners. Hence, in the twenty-first century and beyond, the requisite knowledge and skills of trainee doctors should not be limited to clinical medicine.

Allocating resources to the medical humanities has at times proved a contentious issue. While most medical schools purport to understand the value of the medical humanities to education, there is a shortfall of teaching staff trained to deliver high quality content (Zhang 2024). Additionally, investment into resources and facilities that improve teaching of medical humanities is overshadowed by the money put into clinical education (Zhang 2024). This means that course structure differs between each medical school. However, depending on staff and investment, quality is highly variable. Since it is difficult to balance these two aspects of medical education, medical humanities may often be taught more superficially, due to time constraints (Wachtler et al. 2006; Thacker et al. 2022; Zhang 2024). Many medical schools may offer elective subjects to students, but this is highly dependent on student preference and advertising by administrative staff (Wachtler et al. 2006; Assing Hvidt et al. 2022; Zhang 2024).

### International context

While teaching on medical ethics is nearly universally included in medical education curricula, the same cannot necessarily be said for the medical humanities more broadly. The American Association of Medical Colleges (AAMC) have endorsed the integration of the arts and medical humanities into medical school curricula since it can “effectively contribute to optimal health care outcomes for patients and communities” (Howley et al. 2020). The first medical school to include the medical humanities in their curriculum after the Flexner report was Case Western Reserve University School of Medicine in 1952 (Klugman 2018).

Since then, an analysis performed by Howick et al. (2022) showed that 75% of medical schools in the United Kingdom and the United States offer the medical humanities in their curriculum, while half of Canadian medical schools did the same. However, only a third of medical schools in the United States and Canada and 10% in the United Kingdom have compulsory content (Howick et al. 2022). For medical schools in the United States, most of the medical humanities teaching is integrated into the degree with there being some scope for elective coursework.

Generally, medical schools have transitioned towards incorporating the medical humanities into the degree as opposed to standalone electives, due to ease of delivery and satisfaction of course criteria. However, this may decrease the actual content that students are exposed to, with potential for medical humanities teaching to “get lost” in the curriculum. While elective subjects aim to combat this, uptake is often poor as a result of students opting for further clinical time or poor scheduling and investment from medical schools (Klugman 2018). Of note, studies have demonstrated a correlation between greater medical humanities content and lower medical school ranking (Howick et al. 2022). Possible explanations for this phenomenon include lower ranked schools being more receptive to improvements, and conversely, higher ranked schools embracing the tradition and apparent success of established curricula (Howick et al. 2022).

In mainland Europe, all medical schools have humanities content in their course. The history of medicine and bioethics are present at most universities, but courses exploring literature, visual arts and sociology are minimal (Orefice et al. 2019). Individual subjects that further explore the humanities exist but are more common in Spain than Italy (Orefice et al. 2019). In Denmark, medical humanities classes are compulsory, with additional scope for students to do further study in an elective capacity (Assing Hvidt et al. 2022).

In China, most medical schools have compulsory education in the medical humanities as well as elective courses (Kosik et al. 2014). Methods used to teach students focus on movies and photography as opposed to reading and writing seen in Western medical schools (Kosik et al. 2014). Classes decrease in each subsequent year of training due to clinical placement requirements, but this is common practice at medical schools worldwide (Kosik et al. 2014).

Assessing the effectiveness of different methods of teaching the humanities is difficult. Gender, age, medical school location and prior study can influence interest and outcomes (Schwartz et al. 2009). A meta-analysis by Zhang et al. (2023) found no significant difference between reflective writing and clinical practice interventions on improving empathy. Further, in some settings, longer medical humanities programs were associated with decreased student empathy (Zhang et al. 2023). Hypotheses for this finding include students gradually becoming bored, study burnout and empathy fatigue.

### Australian context

While the extent to which medical schools incorporate the medical humanities is highly variable, Australian medical schools have similar approaches to North America and Europe. All undergraduate and postgraduate degrees have medical humanities embedded into the curriculum, often with scope for further elective coursework. Most universities also take advantage of problem-based learning classes so students can practice viewing patients through a holistic lens.

## University of Melbourne PP curriculum

The Melbourne Medical School Professional Practice curriculum has had a presence in the medical program for more than 15 years. Initially as Empathic and Ethical practice (EP), Professional Practice (PP) now primarily focuses on content addressing the Australian Medical Council Graduate Outcome Domain 2: “Professional and Leader” (AMC 2023). The key themes in this vertically integrated curriculum include reflective practice, collaborative practice, leadership, advocacy, professional identity formation, medical ethics and law. This content is delivered in one-hour, weekly (or fortnightly in final year) tutorials. These occur as face-to-face tutorials in the first two years of the course, and online via videoconferencing in the final two years.

A redesign of the PP curriculum, commencing in 2019, emphasised two pedagogical underpinnings: workplace learning and medical humanities. Although PP occurs outside of clinical time, the intention is that curriculum content is tied to student’s workplace learning, encouraging critical engagement with these clinical experiences (Billett et al. 2018). Secondly, by underpinning PP with medical humanities, the curriculum sought an explicit focus on the human experience; the students’ own and the lived experience of others involved in healthcare (patients/carers, colleagues etc.). Tying PP to the humanities sought to develop students professionally and personally alongside a more traditional biomedical curriculum (Kumagai 2017).

In addition, during the curriculum re-design process it was noted that students’ engagement significantly increased, during sessions where they or their peers were required to present on a given topic. Indeed, many tutors reported applying this method informally during tutorials. Resultantly, this was formalised into the concept of co-facilitation, with a guiding principle that this would allow tutors to gradually ‘recede’ across the course of the four years as the students attained more independence and responsibility for content delivery.

## Methods

The following student reflection was devised in collaboration between a student enrolled in the PP program, one of the course tutors, and two course designers, to explore student experiences of the medical humanities in medical school curriculum. Intentional effort was made to focus on student perspectives of PP and contextualise these within the broader literature. Points of discussion included overall student views of PP, utility of assessments and key experiences. However, the central focus was on students’ engagement with the coursework, and in particular the co-facilitation process, which forms an integral part of the course in senior years. While best efforts were made to capture the overall impression of the cohort, this reflection ultimately represents an individual student’s experience. Additionally, experiences are likely to be diverse, and will be influenced by clinical placement site and cohort, due to differences in exposure to real ethical dilemmas, clinical exposure and teaching staff. Nonetheless, the reflection provides a unique perspective capturing current opinions of the medical humanities amongst medical students.

## Student perspective of professional practice

Students are acquainted with the PP program at the University of Melbourne throughout the entire duration of the Doctor of Medicine degree. It is one of the only threads that runs through the whole degree. As classroom problem-based learning and clinical skills tutorials are replaced with ward-based learning, PP remains a consistent presence. It is clear that the medical school values and prioritises the medical humanities in students’ learning. However, for some students this may be at odds with preconceived expectations of what medical study entails (Assing Hvidt et al. 2022).

PP is considered by some students as a subject that requires minimal effort and provides minimal gain. It is through this lens that some students may view medical humanities coursework as a “waste of time”; why should I learn about the humanities when I should be studying the clinical content that will help me to become a better physician? Common criticisms are that certain discussion topics—creativity, flourishing, self-compassion—do not feel relevant to the medical course and that there should be a singular focus on medical ethics. In particular, a common thread through all years of PP that students often find challenging are freeform tutorials where students can reflect on their journeys through medicine as a collective. This may include anecdotes from placement, ethical quandaries that require further elaboration or simply a space to talk about anything on one’s mind. However, while some students may view the PP curriculum through this unproductive lens, learning the medical humanities should be an immersive experience that provides a necessary break from didactic teaching. With clinical content including lectures, tutorials and placement forming the majority of contact hours, PP serves as a “circuit breaker” from this stress.

PP can at times present students with unfamiliar learning and social environments, where an expectation to discuss challenging content and share vulnerably within a “brave space” is created (Arao and Clemens 2023). While there is often a natural discomfort in being vulnerable amongst peers, it is the flexibility of these classes that often generates such uneasiness. In a course that frequently requires strict adherence to a timetable and an emphasis on structure, it can feel foreign to interrogate ideas in a space where rigidity is avoided and uncertainty embraced. Despite this, it is through discussion of these themes that students shape and develop skills of understanding, compassion and reflective practice. For example, one of the threads throughout the course that is regularly revisited is engagement with transdisciplinary teaching (Thacker et al. 2022). This is primarily achieved by cross cultural exploration and understanding of First Nations Health, introducing students to themes including self-determination, cultural awareness and cultural safety which are central to understanding and optimising care for Australian Aboriginal and Torres Strait Islander Peoples. Additionally, while not part of the main PP program, the University has introduced the “Narrative Medicine” elective which allows for further interaction with the humanities through storytelling and regular museum and gallery visits. Together, these initiatives are ways in which the medical humanities upend the rigid structure that students are familiar with by exploring the subjectivity of the human condition (Bleakley 2023). However, it is these ideas that are required to become a professional practitioner that emphasises “caring medicine” over a “curative” focus (Bleakley 2015).

Written reflections and oral presentations form the basis of assessment in PP. While any assessment may induce feelings of anticipatory dread and irritation for some, often assessments in PP seem to create the most hostility in the student body. Generally, medical students think that there are too many assessments and that they do not contribute greatly to their development as students (Petrou et al. 2021). However, assessments within PP are sometimes difficult to translate to the standard format that students are familiar with. In a world of multiple choice questions and clinical examinations, expressing creativity in a flexible manner through writing is at odds with the structured dogma that is ingrained in much of medical education. De la Croix and Veen (2018) contend that if assessments are deemed unimportant, students will not fully participate in them and will do the minimum to pass, defeating the purpose of these tasks. One method to address this barrier is to grade students based on participation in class discussions, but this too has its limitations (Petrou et al. 2021). Overall, effective messaging on the utility of assessments, combined with engaging tasks that do not add to the mundanity of testing medical knowledge, will aid in greater appreciation of their importance.

In their first year, students engaged with cultural artefacts as a way of introducing the complexity of the human experience and its link to patient-centred care (Harris et al. 2024). The first weeks of the program are marked by presentations of personal artefacts that represent the idea of a “good doctor”. Many of the artefacts presented, such as photos of family members, heirlooms or books, catalysed discussions around mortality; students often highlighted the importance and privilege of providing optimal end of life care whilst also recognising its inevitability in future practice. Consequently, there was space to exercise compassion in these sessions which has been hypothesised to help grapple with and approach death in clinical medicine (Harris et al. 2024). Students also participated in Pecha Kucha, a style of presentation consisting of 20 slides that automatically progress after 20 s (Beyer 2011). Each slide contains one photo or artefact that underscores learnings and development from the year, with the presenter explaining its significance and reason for selection. Presentations provided insights into the experiences and challenges throughout the year with topics such as imposter syndrome and motivation. Students also appreciated the vulnerability expressed by peers as this reinforced their growing engagement with important ideas of subjectivity and individuality.

In the second year, the patient-partner program revealed how the doctor-patient relationship must be viewed from a holistic lens, rather than a purely medical one. The main reflection from students centred around the importance of interdisciplinary care and its positive impact on patient care. While some patients unfortunately passed away during this time, honest discussions around death and the value of pastoral care were a highlight for many. As medical students may receive limited exposure to religious and spiritual themes on placement, this proved to be a valuable insight into topics that will be frequently encountered at the bedside.

In the third and fourth years, students are expected to take turns leading tutorials, and this presents new challenges for students. Co-facilitation involves a different student being selected weekly to lead the tutorial on an assigned topic. Part of the preparation included reading over the provided materials and navigating the discussion with the small group tutor throughout the hour long session. Students were not provided any training on how to co-facilitate nor informed of current pedagogy on how to approach tutorials. This allowed for a more organic process that encouraged students to trial co-facilitation styles without pressure. Since there were many tutorials throughout the year, students also had multiple opportunities to learn from how other students co-facilitated, and could apply these strategies in future sessions. This approach made the experience less drily academic, and reinforced the importance of problem solving and engagement in effective communication.

The main highlight of this process was being able to generate interesting, in-depth discussion around topics that can sometimes be quite uncomfortable to talk about. This awkwardness comes from students’ lack of experience in areas that may require thoughtful consideration. Clunky phrasing, “word blanking” and challenges in articulating an opinion or position were common student experiences during co-facilitation. Additionally, students’ innate desire for accuracy and truth often made for fascinating experiences of discordance in situations or scenarios where a singular “right answer” may not exist. Often, students found themselves in situations where they found it difficult to know how to best express their thoughts. Hence, being a co-facilitator meant that there was added responsibility to prepare for these discussions and as a result, improved students’ ability to form and develop cogent thoughts about areas they were less familiar with.

One of the key experiences shared by many students over the course of the year was the apparent tension between “gold standard” medicine that is taught, and “real world” medicine practiced at the bedside. Whether it be insensitive styles of patient interaction or questionable collegiality with fellow members of the treating team, communication challenges can shake the core of a medical student experience: what truly is the ideal doctor? Does work burn you out to the point of neglecting these concerns? Regardless of the answer, insights like these are only possible once the group has cohesion, with the co-facilitator playing a pivotal role in this. The co-facilitator’s role is to create a safe space that fosters a policy of inclusion without judgement.

The overarching challenge of the co-facilitation process was taking the initiative to participate. Generally, low participation from one’s peers may be a common experience for co-facilitators and tutors alike. There are a variety of reasons for this, including lack of engagement with the topic, boredom and fatigue from a day of placement and classes. Online sessions delivered via videoconferencing, as opposed to face-to-face tutorials, may also contribute significantly to a lack of engagement (Nesher Shoshan and Wehrt 2022). Formulating a teaching style for the session also proved to be difficult for some students. With no fixed example to follow, there was a tendency to model one’s approach based on how others decided to tackle the role. One strategy implemented by some students was to call on individuals to contribute directly. This method facilitated the inclusion of all students, while mitigating the awkwardness of talking excessively or over someone. By giving students the space and a direct invitation to contribute, this reduced the anxiety that some students felt in “speaking up”. While calling on people to contribute can be antagonistic to organic discussion, the weekly connection that students develop with each other can offset some of the inherent anxiety with being put on the spot. Additionally, sessions occurring online may further decrease the intimidation that students feel from this approach due to a sense of physical detachment. Another common approach to co-facilitation was to use videos as the main driver of discussion. For example, new concepts that were introduced, such as the concept of a “Benefit Mindset” (Buchanan and Kern 2017), were explained with a video that described its meaning and provided a base from which discussion could develop. An approach to co-facilitation that was not as popular was a more didactic, “lecture” style of tutorial delivery, during which the co-facilitator spoke without group interaction for most of the session. This usually came in the form of teaching a concept or summarising readings. While this was perhaps a useful way to digest large amounts of new content, there was an overreliance on the co-facilitator to talk and a lack of group participation or interactivity.

Some sessions were more content heavy than others—an hour of reflection compared with grappling the intricacies and grey areas of medical ethics, for example—but this sits on a spectrum. For some, a structured tutorial with numerous resource materials was preferred to the comparatively unstructured reflective tutorials. Indeed, the unstructured style of reflective tutorials led to some sessions that relied heavily on the tutor or co-facilitator to generate stimulating discussion. However, these conversations replicate many of the philosophical and introspective exchanges that students will have in the future with their patients. PP provides a platform and safe space for students to practice discussing challenging or contentious topics and troubleshoot in real time if things do not go as planned.

Another highlight was seeing students’ confidence grow as the year progressed. Becoming comfortable enough to share deeply personal experiences is not something to be taken lightly. Despite being in the same year and at the same hospital, familiarity between student peers is not necessarily guaranteed. Co-facilitation is more difficult when one is unfamiliar with individual habits and tendencies; when to allow for silence, when to call on someone to speak, when to monologue. However, the insights shared became more meaningful as students grew more acquainted with one another. As clinicians, students will find themselves in situations where they are called upon to lead meetings or conversations without being familiar with everyone in a group. This may be in the form of a family meeting or a multidisciplinary team meeting. Hence, practicing these skills in a controlled environment helps to sharpen teamwork, collaboration and leadership abilities in students’ professional and everyday life.

## Conclusion

The medical humanities and medicine have a complicated yet underappreciated relationship. The benefits of incorporating the medical humanities into medical curricula are well-documented and in a rapidly changing world where the demands for healthcare services are increasing, the challenges faced by doctors require a balance of clinical acumen and emotional intelligence. The skills that the humanities instil in students are crucial in providing effective, patient-centred and culturally safe care. Most medical schools worldwide have the medical humanities embedded into their curriculum. Outcomes such as increased empathy are not significantly associated with mode of delivery but may be negatively affected by length of exposure.

Generally, PP and the medical humanities are treated by many as secondary to clinical knowledge. While this mindset may exist before medical school, it is further reinforced by students’ lack of familiarity and discomfort with the themes covered in these sessions. Furthermore, assessment styles, such as written reflections, that are traditionally used in the medical humanities are unpopular as they do not mirror common forms of clinical assessment. Oral presentation-based assessments can be a valuable catalyst of discussion surrounding important topics encountered as clinicians. Co-facilitation allowed students to develop communication skills, collegiality and leadership in a safe, low pressure environment. It challenged students’ initiative to test different styles of facilitation as well as consider how to best generate student engagement. Styles such as calling on people individually appeared to be more effective than a more didactic approach. However, participation in PP was variable and dependent on fatigue, interest in discussion topics and empathy fatigue.

Overall, shaping students’ perspective on the value of the medical humanities is key to its success. Student perspectives have been shown to be beneficial in optimising engagement with medical humanities curricula (Shapiro et al. 2009; Ahmed et al. 2024). We believe this reflection provides greater understanding of student engagement with themes that may generate scepticism and antagonism. Opinions shared in this article appear to reflect student consensus worldwide about the provision and reputation of medical humanities education (Petrou et al. 2021; Assing Hvidt et al. 2022; Makowska et al. 2022; Olding et al. 2023). Recurrent feelings of unfamiliarity with content delivery and discomfort with assessment styles may raise questions in all medical students regarding the utility of such initiatives. Hence, while most medical schools celebrate its inclusion in the curriculum, current student and faculty attitudes towards programs like PP may prevent the medical humanities from resonating with students. This may have negative effects on the development of empathetic, professional practitioners. It is not only incumbent on the student to be immersed in the humanities, but also on the university to cultivate an environment that integrates it into the curriculum in a way that is engaging and harmonious. Consequently, students may be more likely to elevate the medical humanities to that of equal footing with the traditional biomedical curriculum.

## Data Availability

No datasets were generated or analysed during the current study.
